# Consumer Expectation of Flavored Water Function, Sensory Quality, and Sugar Reduction, and the Impact of Demographic Variables and Woman Consumer Segment

**DOI:** 10.3390/foods11101434

**Published:** 2022-05-16

**Authors:** Uijeong An, Xiaofen Du, Wanyi Wang

**Affiliations:** 1 Department of Nutrition and Food Sciences, Texas Woman’s University, Denton, TX 76204, USA; uan@twu.edu; 2 Center for Research Design & Analysis, Texas Woman’s University, Houston, TX 77030, USA; wwang@twu.edu

**Keywords:** beverage, soft drink, sugar reduction, beverage flavor, refreshing, consumer segment

## Abstract

This study aimed to investigate consumer expectation of flavored water and potential consumer segments. The results showed flavored water was ranked the fourth most popular drink, after plain water, tea, and coffee, by 901 participants. Consumers highly expected functional flavored water with refreshing (87.4% selection), thirst-quenching (73.7%), and tasty (65.7%) qualities, containing vitamins, minerals, and antioxidants, and providing energy. Expected flavored water sensory qualities included temperature (62.4%), flavor (52.4%), and sweet taste (47.4%); lemon, berry, and lime flavors were most preferred, while bitterness, irritation, astringency, and sourness were least preferred. Pure sugar and honey were rated highest as the sweeteners for flavored water. Likewise, consumers were mostly concerned with taste followed by calories. Single demographic variables (age, reported health condition, drinking frequency, educational level) significantly influenced (*p* ≤ 0.05) flavored water function, sensory quality, and sugar reduction expectations. Females had higher expectation of flavored water’s refreshing and antioxidant functions. Cluster analysis revealed two consumer segments. The younger, low-education, self-reportedly less healthy cluster (mainly college students) expected various functions and flavors such as low temperature, cooling taste, diverse flavors, and sweet taste (and disliked bitterness). The older, educated, employed, self-reportedly healthy cluster had lower expectations of flavored water functions, were less sensitive to bitterness, and preferred no sweetness or little sweetness. These findings provide informative data to establish marketing and sales strategies for promoting flavored water.

## 1. Introduction

There are four primary sectors of the global beverages market: soft, hot, milk, and alcoholic drinks [1], while water is usually excluded from beverage categories. Flavored water is enhanced bottled water, ranging from simple flavoring additions to formulations that are equivalent to soft drinks [2]. The flavored water segment started to emerge about a decade ago, as consumers looked to water as a healthy alternative, or simply began to limit carbonated soft drink consumption [3]. Indeed, bottled water shares rose to 22.5% in 2015, taking a market share away from carbonated soft drinks (soda) [4]. Global flavored water market sales reached $10.3 billion in 2018 [4], while the United States had sales of $5.12 billion in the same period [5].

People drink flavored water for hydration and “healthier” properties [1]. The concept of “healthier” drinks includes consumer needs for safety and health benefits. According to an American national survey, healthy choices are most sought after (58%). Of that 58%, 61% utilize water as weight loss and health improvement tools [6]. Half (48%) of American bottled water drinkers also directly substitute water in place of high-sugar drinks [6]. Meanwhile, taste remains a priority, as 39% of Americans list unappealing flavor as reason for low water consumption. Various flavors have been incorporated into water, including lemon, orange, mixed berry, and apple, along with a variety of herbs, vegetables, and spices [7]. Lemon flavor dominates the flavored water market, followed by orange [7]. In addition to diverse flavorings, other major ingredients for flavored water include water, carbohydrates (sucrose or sugar), acidulants, colorings, preservatives, and other functional ingredients [8]. The major functions of these ingredients are to enhance the organoleptic properties, appearance, and stability of the product; and claim certain health benefits. Sugar has several functional properties, including sensory (sweetness, flavor enhancement, and texture) and physical properties as well as many reactions and interactions with other food ingredients present (Maillard reaction and caramelization) [9].

Despite its popularity in the market, no studies have investigated consumer expectation on flavored water, which includes consumption motivation, preference, and the attitude toward sugar reduction. The investigation of consumer expectation on flavored water would aid in establishing a well-rounded understanding of flavored water consumer preference and consumption drivers for marketing in the food industry. Additionally, sugar overconsumption linkage with negative health outcomes, such as cavities, body weight increase, type II diabetes, and cardiovascular disease, make reducing its consumption a globally common health goal [10,11]. The 2010 Dietary Guidelines for Americans recommend limiting the intake of added sugars in the American diet and focusing on consuming nutrient-dense foods and beverages [11]. Sugar-sweetened beverages are a major source of free sugar intake [12]; however, the functional role of sugar makes its removal or reduction difficult. Sugar-reduced foods and beverages remain the most popular and significant trend in the food industry and academic research. Even so, flavored-water product purchase options can range from non-sugared to fully sugared.

Consumer expectation is complex and influenced by multiple variables such as food product intrinsic attributes, consumer genetic variables, consumer demographic information (gender, age, education level), and social determinants (economic, religion, culture) [13]. The influence of these variables can be individual or the interaction of multiple variables. It is important to identify the target consumer segment, therefore, in order to understand consumer needs and wants in addition to establishing marketing and sales strategies [10]. Understanding the preference patterns in various demographic, attitudinal, and usage segments is important for positioning products and identifying niche opportunities in defined markets, although there is no such study for flavored water. In addition, understanding consumer segment and consumer expectation in sugar-reduced beverage/flavored water would provide data support for sugar-sweetened beverage reduction policies.

This study aimed to investigate consumer expectation regarding flavored water functions, sensory quality, and sugar reduction, as well as the impact of consumer single variables (gender, age, health status, consumption frequency, education level, and employment status) and consumer segments (multiple demographic variables). The data collection had a focus on females, while a statistical effectiveness between genders was still maintained.

## 2. Materials and Methods

### 2.1. Participants

All consumer study procedures were reviewed and approved by the Texas Woman’s University (TWU) Institutional Review Board (IRB). Participants for the survey were recruited using a TWU email list to advertise and deliver the questionnaire to university students, faculty, and staff at three campuses (Denton, Houston, and Dallas, TX, USA); TWU participants also forwarded the email to non-TWU individuals. Participants voluntarily took the survey, and the only discriminating factor was the consent age of 18. The survey was administered on the Internet using Google Forms. Questionnaire completion time was designed to be 10–15 min, although participants were instructed to take as much time as needed. Participants were compensated with a cash honorarium for participation.

To obtain a representative sample, the sample size was computed based on 99% confidence interval and 4–5% margin of error. By considering additional 20% survey incompletion or invalid cases, the estimated survey respondents were from 815 to 980.

### 2.2. Survey Design

Nine questions related to flavored water/beverages and six demographic questions were included in this survey (Table 1). Participants were asked to answer the questions related to their expectation and experience of flavored water and beverage consumption. As shown in Table 1, the first three questions were designed to examine flavored water consumption motivation, another three questions were related to consumer specific expectation on sensory quality of flavored water, and the third three questions were for consumer attitude toward sugar reduction in flavored water. Single-response questions and multiple-choice questions (CATA, check-all-that-apply) were included.

Demographic information was collected at the end of the survey, which included questions related to gender, age, reported health condition, flavored water drinking frequency, education level, and employment status, as shown in Table 2. Demographic questions were designed with single-choice answers.

### 2.3. Statistical Analysis

The frequencies and percentages of answers from the survey were first obtained using Google Forms. Then, differences within single consumer demographic variables (gender, age, drinking frequency, reported health condition, education level, and employment status) toward nine survey questions were analyzed with chi-square tests. Correspondence analysis (CA) is a multivariate statistical approach used to visualize relationships between the rows and columns of the contingency table for categorical variables. CA analysis is similar to conventional discriminant analysis (DA) [14,15]. In this study, CAs were performed to visualize the relationships between each demographic variable and its response to nine survey questions related to flavored water; only squared cosines > 0.5 (indicating the importance of factor for each category of the survey questions) were retained in the analysis [16]. The relationships were inferred from the variables’ positions in the shared geometric space generated from CA and defined by chi-square distance.

Consumer segment demographical pattern analysis (pattern recognition of six demographic variables) was performed with hierarchical cluster analysis (HCA) to group consumers with similar flavored-water preferences according to 61 attributes over the nine survey questions. Ward’s method was used in the HCA to group the observations based on the reducing sum of squared distances of each observation from the average observed value in a cluster. This method began by separating every participant into a cluster by themselves. At each stage of the analysis, the two most similar cluster/participants were linked until all the participants were joined in a complete classification tree. Pearson’s chi-square tests were used to compare differences in flavored water preferences between the two grouped clusters. The same analyses were also used to compare the demographics between the two grouped clusters. Multiple correspondence analysis (MCA) was used to visualize the relationships between clusters and multiple demographics with 901 observations (rows) and 6 columns of demographics plus 1 cluster column. This method allowed the simultaneous, visual evaluation of the association among multiple nominal variables. Therefore, the associations between clusters and demographics were interpreted by chi-square tests and visualized in MCA.

Internal preference mapping (IPM) generated from principal component analysis (PCA) was subsequently conducted on flavored water preference by clustering to further visualize and validate the HCA results in terms of grouping consumers. IPM can derive a multidimensional representation of flavored water preferences and consumers in order to help identify what product preferences correspond to what cluster of consumers. By this step, an IPM was formed to characterize consumer segments. Due to the large number of items for the flavored water preferences, only the items showing significant relationships with the clusters in the chi-square tests were included in the IPM [16].

All statistical tests assumed a significance of α ≤ 0.05. Chi-square tests were performed in SPSS (version 25, IBM, Armonk, NY, USA), and all the other analyses and visualizations (CA, HCA, IPM, MCA) were conducted in XLSTAT (version 2019, Addinsoft, New York, NY, USA).

## 3. Results

### 3.1. Demographic Information and Data Treatment for Statistical Analysis

A total of 906 signed for the survey, while 901 participants completed this survey (Table 2). Among them, 91.3% (*n* = 823) were female, while males accounted for 8.4% (*n* = 76). The survey had an unbalanced gender ratio, with an original design to investigate women consumers. Women consumers are the major purchaser for flavored water (private communication). On the other hand, a sample of 76 male participants is considered high for a consumer study and effective for a statistical gender comparison.

As shown in Table 2, the distribution of age groups was 48.8% (*n* = 440) for 18–25 years old, 17.9% (*n* = 161) for 26–30 years old, 10.8% (*n* = 97) for 31–35 years old, 5.3% (*n* = 48) for 36–40 years old, 4.8% (*n* = 43) for both 41–45 and 46–50 years old, and 7.7% (*n* = 69) for 51 years and older. Many more young people participated in the study, implying that the participants represented a general population consuming flavored water. For the purpose of statistical analysis, four age groups (36–40, 41–45, 46–50, and 51 and older) were combined and treated as one group (36 and older).

Participants’ health condition was self-evaluated (Table 2). Very healthy, healthy, and somewhat healthy accounted for 7.7% (*n* = 69), 43.5% (*n* = 392), and 41.0% (*n* = 369); while unhealthy and very unhealthy accounted for 6.8% (*n* = 61) and 1.1% (*n* = 10). The majority of the participants (>90%) had health conditions. For statistical analysis, two groups (very healthy and healthy) were combined to a single group as “healthy”, while another two groups (unhealthy and very unhealthy) were also combined to a single group as “unhealthy”.

Participants’ flavored water drinking frequency included 43.1% (*n* = 388) for heavy drinkers (several times a week), 28.3% (*n* = 255) for moderate drinking (1–4 times per month), 4.9% (*n* = 44) and 19.5% (*n* = 176) for less frequency (less than once a month and only occasionally), and 4.2% (*n* = 38) for never consuming flavored water. Overall, 71.4% of the participants were shown to drink flavored water/beverages at least once per month; therefore, the participants recruited for this study represented a group who had high consumption frequency and familiarity with the product type tested in this survey.

The participants’ education level showed 72.2% of the participants had an associate degree or higher, namely 9.3% (*n* = 84) for doctoral, 20.2% (*n* = 182) for master’s, 30.2% (*n* = 272) for bachelor’s, and 12.5% (*n* = 113) for associate. Around 23.9% (*n* = 215) of the participants had some college education without a degree, while only 3.9% (*n* = 35) graduated from high school or lower. Since this survey form was mainly sent out to the TWU email accounts, overall education might be higher than the average US population. For statistical analysis, two groups (some college without degree and high school or lower) were combined to a single group of “some college without degree or lower”.

Participant employment status was diverse. College students accounted for 37.2% (*n* = 335), while 36.5% (*n* = 329) and 21.4% (*n* = 193) claimed to have full-time and part-time jobs. The remaining participants were self-employed (1.1%, *n* = 10), homemakers (1.2%, *n* = 11), unemployed (2.1%, *n* = 19), or retired (0.4%, *n* = 4). For statistical purposes, the last four small groups (unemployed, self-employed, homemaker, and retired) were combined and treated as a single group called “unemployment”.

### 3.2. Flavored Water Popularity and Consumption Motivation

**Figure 1:**
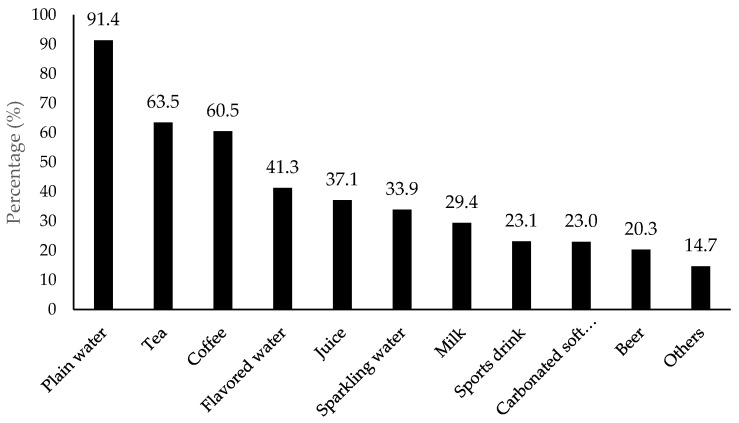
Percentage (%) for type of drinks/beverage that consumers drank the most (*n* = 901). Others included soda, diet soda, wine, coconut water, kombucha, and non-dairy milk according to the collected data.

According to the survey results for commonly consumed drinks (Figure 1), plain water received the highest amount of selection by consumers, up to 91.4%, followed by tea (63.5%), coffee (60.5%), and flavored water (41.3%). Plain water was out-ranked over other beverages, while flavored water was ranked in the top 4 most popular drink selections out of 10 drink/beverage options. Results also implied that flavored water was an important and major product in the beverage market, which has been addressed by other researchers [1,2].

**Figure 2:**
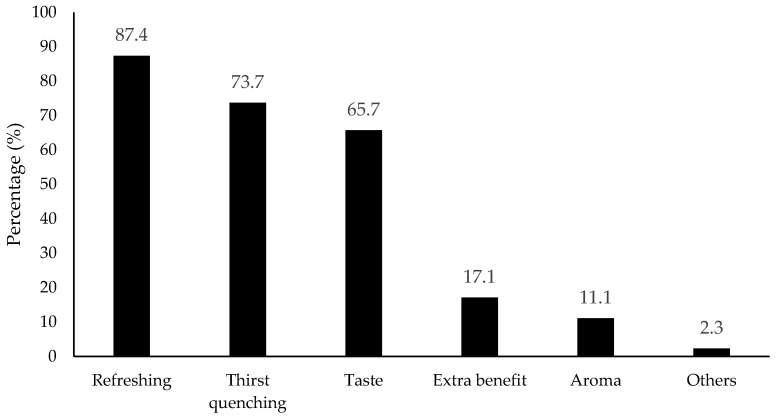
Percentage (%) for the effects that consumers expected the most from the flavored water/beverage (*n* = 901). Others included health, low calories, headache relieve, body hydration, and electrolytes according to the collected data.

The motivations for flavored water consumption were tested by two questions, as shown in Figure 2. Consumers most commonly expected a refreshing effect (87.4% selection), followed by thirst-quenching (73.7%) and taste (65.7%). It should be noted that the concept and application of refreshing perception for foods and beverages is rather unexplored.

**Figure 3:**
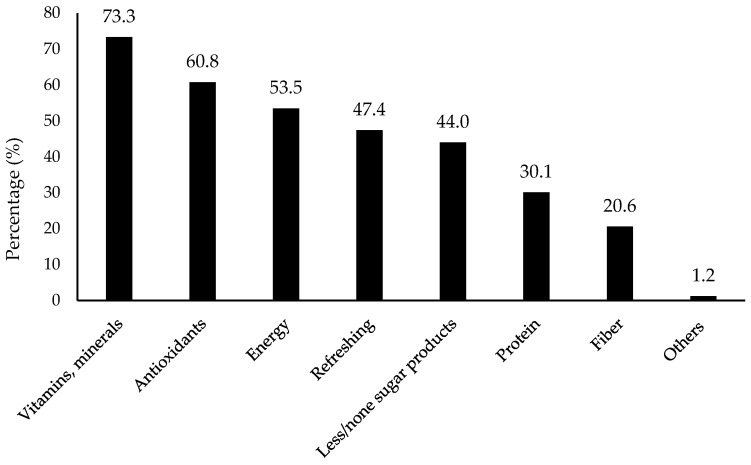
Percentage (%) for extra benefits that consumers expected to be added (*n* = 901). Others included grapefruits, strawberry, orange, passion fruit, acai, coconut, herb, pomegranate, cucumber, vanilla, and ginger according to the collected data.

As shown in Figure 3, the expected extra benefits to be added for flavored water included vitamin and minerals (73.3% selection), antioxidants (60.8%), energy (53.5%), refreshing (47.4%), and less and/or no sugar (44%). It further showed refreshing as one of the major factors that consumer considered for flavored water consumption.

### 3.3. Expectation on Flavored Water Sensory Quality

**Figure 4:**
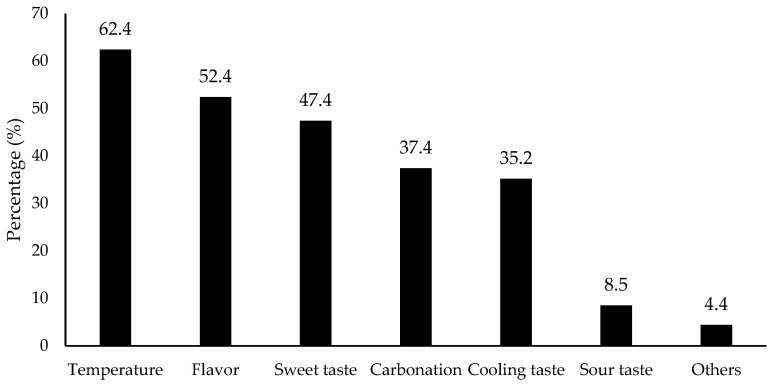
Percentage (%) for the factors that consumers considered the most when deciding to drink flavored water/beverages (*n* = 901). Others included caffeine, sugar free, thirst-quenching ability, calories, price, convenience, nutrition facts, artificial taste, health benefits, and organic ingredients according to the collected data.

As with the aforementioned consumer expectation of taste (Figure 2), sensory qualities determining consumers’ choice of flavored water were further investigated in this study. As shown in Figure 4, temperature (62.4% selection) was shown to be the most effective factor, followed by flavors (52.4%), sweet taste (47.4%), carbonation (37.4%), and cooling taste (35.2%). Refrigerator temperature (4 °C) is a common beverage consumption temperature.

**Figure 5:**
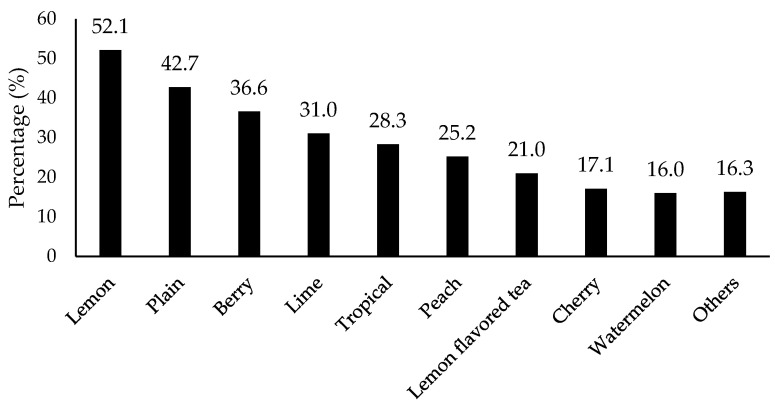
Percentage for the types of flavors for flavored water/beverages that consumers drank the most (*n* = 901). Others included orange, grapefruit, raspberry, strawberry, blackberry, pineapple, mango, pomegranate, grape, apple, kiwi, fruit punch, passion fruit, cucumber, mint, vanilla, coconut, and cola according to the collected data.

**Figure 6:**
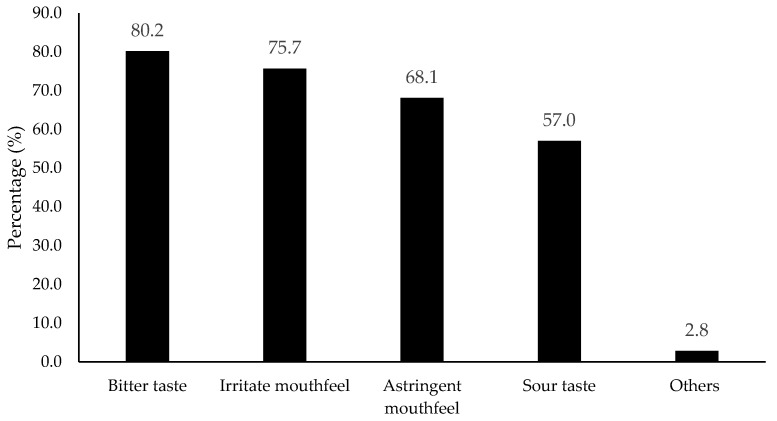
Percentage (%) for the attributes that were not expected from flavored water/beverage (*n* = 901). Others included carbonation, sweet, artificial flavors, lingering taste, and chemical taste according to the collected data.

Preferred specific flavors were asked and results are shown in Figure 5. Lemon (52.1% selection), plain (non-flavored, 42.7%), berry (36.6%), and lime (31.0%) were the top four flavors. The results indicate citrus family flavors were more preferred than others, most likely due to consumer familiarity. In contrast, all bitter (80.2% selection), astringent (68.1%), irritate (75.7%), and sour tastes (57%) were considered as negative attributes that consumers did not expect for flavored water (Figure 6).

### 3.4. Consideration on Choosing Sugar-Reduced Flavored Water

**Figure 7:**
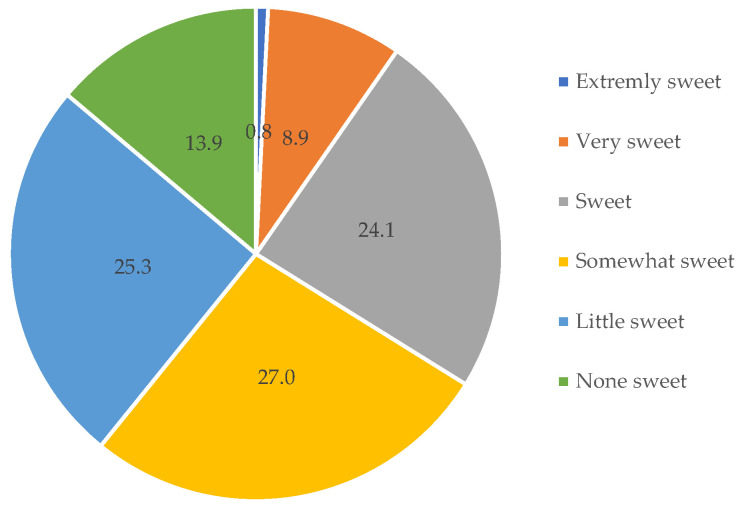
Percentage (%) of sweetness liking of the regular beverage for consumers (*n* = 901).

This study found the consumer preference on sweetness perception of flavored water to be evenly distributed (Figure 7). Somewhat sweet (27.0% selection), little sweet (25.3%), sweet (24.1%) and non-sweet (13.9%) showed a similar preference, while very sweet (8.9%) and extremely sweet (0.8%) received far fewer responses. The results indicated that consumers preferred the sweetness taste from “non-sweet” to “sweet”, but no more than “very sweet”.

**Figure 8:**
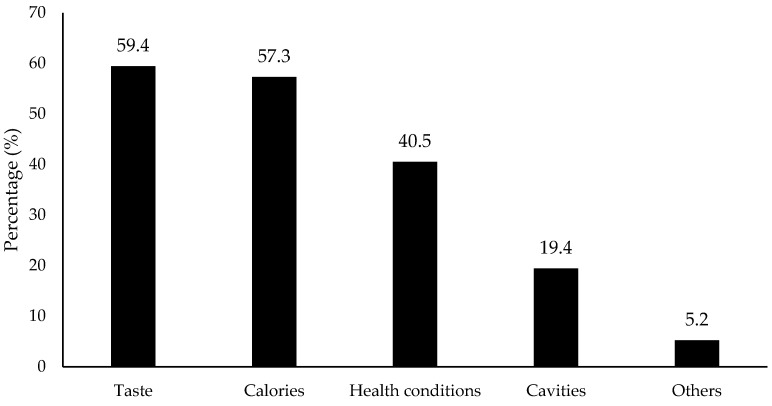
Percentage (%) for the factors that consumers thought were the most important when they chose sugar-reduced products (*n* = 901). Others included alternative sweeteners, natural ingredients (artificial sweeteners), and carbohydrates according to the collected data.

The specific factors that consumers consider for the reduced-sugar flavored water were taste (59.4% selection), calories (57.3%) and their health conditions (40.5%), as shown in Figure 8. The results further confirmed the taste or flavor was the most important factor to determine consumption.

**Figure 9:**
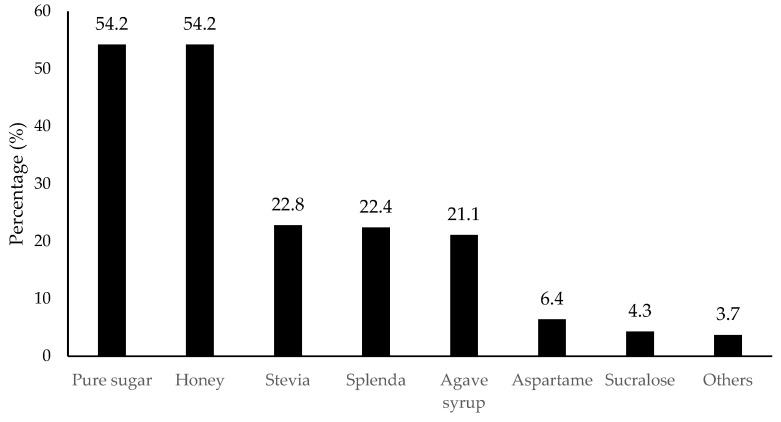
Percentage (%) for the preference of sweeteners (*n* = 901). Others included fructose, monk fruit, saccharine, high-fructose corn syrup, maple syrup, dextrose, and fruit juice.

In terms of the choice of sweetener, as shown in Figure 9, consumers preferred natural sweeteners more, such as pure sugar (54.2% selection), honey (54.2%), and stevia (28.8%), compared to artificial sweeteners such as aspartame (6.4%) and sucralose (4.3%). The results were consistent with the current trend in natural sweetener preference.

### 3.5. Impact of Single Demographic Variables—Gender, Age, and Reported Health Condition

Both chi-square analysis and correspondence analysis (CA) were conducted for each demographic variable. CA did not display a gender difference (*p* = 0.253) toward the nine survey questions overall, while chi-square tests indicated differences between the responses for some survey questions from females and males. The analyses did not find significantly different gender preferences toward flavored water or other popular drinks such as plain water, tea, and coffee. Although the refreshing function expectation of flavored water was rated the highest for all participants, gender was a factor differentiating this expectation as females showed significantly higher (*p* = 0.039) expectation. Other expectations such as thirst-quenching and the taste of flavored water did not show difference between genders. In the expectation of flavored water’s additional benefits, more females expected antioxidant functions, although there was no difference in expectation for vitamins and minerals.

**Figure 10:**
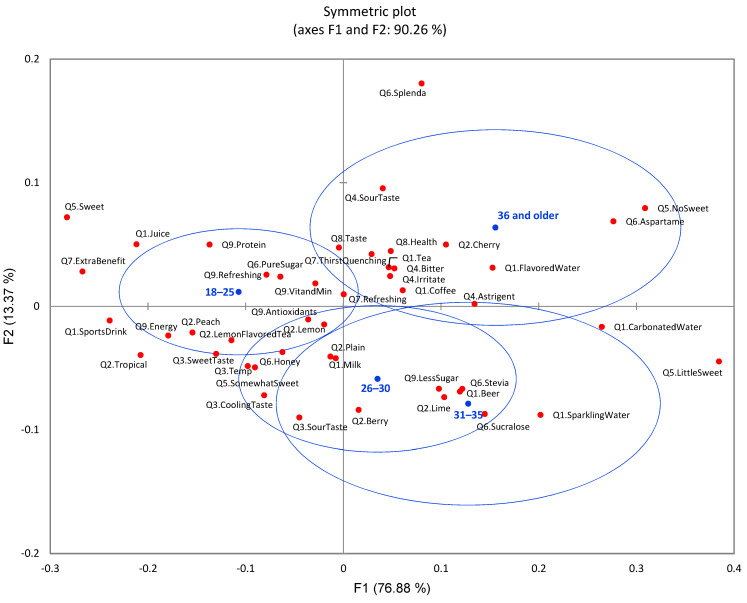
The first two dimensions of correspondence analysis (CA) symmetric plot using age (18–25, 26–30, 31–35, and 36 and older) as rows and all nine survey questions as columns (*n* = 901) (4 rows × 54 columns). Confidence ellipses on the plot were automatically created by XLSTAT software based on CA with observed *χ*^2^ = 317.89 and *p* < 0.0001.

CA showed a significant difference (*p* < 0.0001) among age groups with the nine survey questions (Figure 10). Plain water did not show a significant difference among different age groups, while age groups showed significant preference differences toward other drinks such as flavored water, tea, and coffee. The younger generations, aged 18–30 years old, preferred sports drinks and juice more, while the aged group (31 and older) preferred flavored water, sparkling water, tea, coffee, and carbonated water. Considering the sensory quality of flavored water, participants in the 18–30-year-old group considered temperature, cooling taste, and sweet taste as major factors to enjoy flavored water compared to other age groups. The 18–30-year-old group also displayed a significant difference toward specific flavor types. They preferred flavored water with topical, peach, and lemon tea flavors. Older age groups (especially 36 and older) had more concerns with bitter, astringent, and irritate taste and mouth sensations. Additionally, younger generations preferred sweet and somewhat sweet with pure sugar and honey as sweeteners, while older aged groups preferred little sweetness and no sweetness with aspartame, sucralose, and stevia as sweeteners.

**Figure 11:**
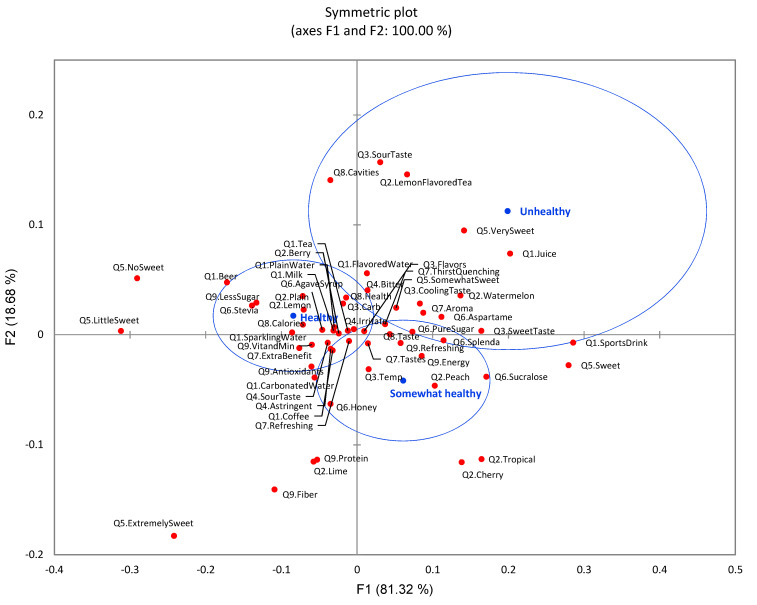
The first two dimensions of correspondence analysis (CA) symmetric plot using health condition (unhealthy, somewhat healthy, and healthy) as rows and all nine survey questions as columns (*n* = 901) (3 rows × 58 columns). Confidence ellipses on the plot were automatically created by XLSTAT software based on CA with observed *χ*^2^ = 208.26 and *p* < 0.0001.

Participant health condition was compared using CA and significant differences (*p* < 0.0001) among three health groups were identified in drink choice (Figure 11). The healthy group had higher preference towards plain water, tea, coffee, beer, milk, sparkling water, and carbonated water, while unhealthy and somewhat healthy groups preferred sports drinks and juice. The unhealthy group had a higher demand for thirst-quenching function, energy, and flavor from flavored water. In contrast, the healthy groups placed higher expectations on refreshing function, extra benefits, vitamin and mineral, antioxidant, and less sugar. Regarding expectation of sensory qualities of flavored water, unhealthy groups consistently showed that flavor, cooling taste, and sweet taste were important for them. They preferred watermelon, tropical, cherry, and peach flavors, while the healthy group preferred plain, lemon, and berry flavors. Furthermore, the unhealthy group claimed that they had less concern about sour and astringency compared to the healthy group. For the sugar content in flavored water, the healthy group preferred no sweetness and little sweetness with concern of calories. The healthy group’s sweetener preference was stevia and agave syrup. In contrast, the unhealthy group preferred sweet and very sweet with regard to the taste of flavored water. The unhealthy group preferred pure sugar, sucralose, and aspartame as sweeteners.

### 3.6. Impact of Single Demographic Variables—Flavored Water Drinking Frequency, Education Level, and Employment Status

**Figure 12:**
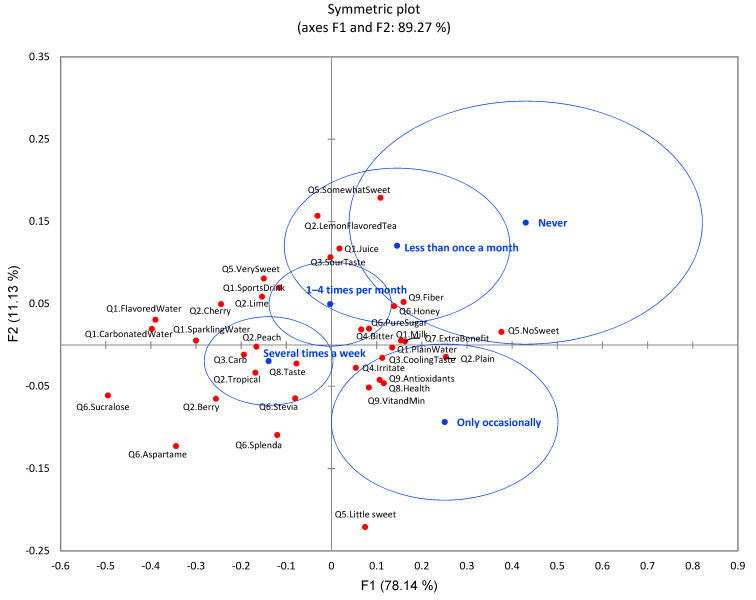
The first two dimensions of correspondence analysis (CA) symmetric plot using drink frequency (never, only occasionally, less than once a month, 1–4 times per month, and several times a week) as rows and all nine survey questions as columns (*n* = 901) (5 rows × 37 columns). Confidence ellipses on the plot were automatically created by XLSTAT software based on CA with observed *χ*^2^ = 414.84 and *p* < 0.0001.

With CA, significant differences (*p* < 0.0001) were identified among the five drinking frequency groups (Figure 12). Participants with high drinking frequency (several times a week) of flavored water/beverages consumed more flavored water, sparkling water, carbonated water, and sports drinks, while the less drinking frequency group consumed more plain water and milk. The low-drinking-frequency groups (never, only occasionally, and less than once a month) had higher expectation of extra benefits of flavored water, and these expectations were reflected in their choice of vitamin and mineral, antioxidants, and fiber content in flavored water. In terms of flavored water sensory quality, the high-drinking-frequency group preferred carbonation with lime, tropical, berry, cherry, and peach flavors. This group had less concern with bitterness taste and irritating mouthfeel of flavored water. In contrast, the low-drinking-frequency groups preferred plain and had more concerns about bitterness taste and irritate mouthfeel. These results were coincident with the low-drinking-frequency groups’ plain water preference. Considering sugar reduction in flavored water, the higher-drinking-frequency group preferred very sweet flavored water with artificial sweeteners such as sucralose, aspartame, and stevia. Taste was a key factor for them to consider when choosing sugar-reduced flavored water. In contrast, the low-drinking-frequency groups preferred flavored water which was not sweetened. The low-drinking-frequency groups listed natural sweeteners such as pure sugar and honey as their preference, since health was a key factor for them in choosing sugar-reduced flavored water.

**Figure 13:**
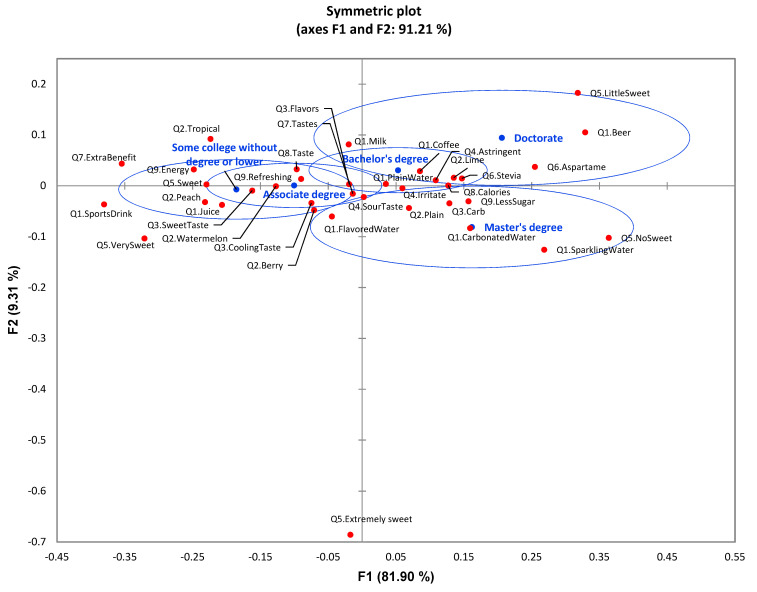
The first two dimensions of correspondence analysis (CA) symmetric plot using education level (some college without degree or lower, associate degree, bachelor’s degree, master’s degree, and doctorate) as Rows and all nine survey questions as Columns (*n* = 901) (5 rows × 44 columns). Confidence ellipses on the plot were automatically created by XLSTAT software based on CA with observed *χ*^2^ = 375.26 and *p* < 0.0001.

Using CA, significant differences (*p* < 0.0001) among five education groups were identified. As shown in Figure 13, the higher education groups (doctorate and master) indicated a preference of plain water, coffee, beer, sparkling water, and carbonated water, while the lower education level groups (associate or without a degree) had higher preference toward sports drinks and juice. The less educated groups expected flavored water to provide extra benefits, more energy, and refreshing functions, while the groups with higher educational degrees expected less sugar in flavored water. Regarding specific sensory qualities of flavored water, the lower educated groups showed a higher demand on sweet and cooling tastes and some specific flavors (tropical, peach, watermelon, and berry). In contrast, higher educated groups showed a higher demand on carbonated taste and specific flavors of lime or plain. They were concerned with astringent and irritate mouthfeel. Attitude toward sugar and sugar reduction also differed according to education level. The low degree groups preferred very sweet and sweet flavored water, displaying less concern about calories. In contrast, the high degree groups preferred little sweet and no sweet flavored water. This group showed higher concerns on calories and sweeteners of aspartame and stevia.

**Figure 14:**
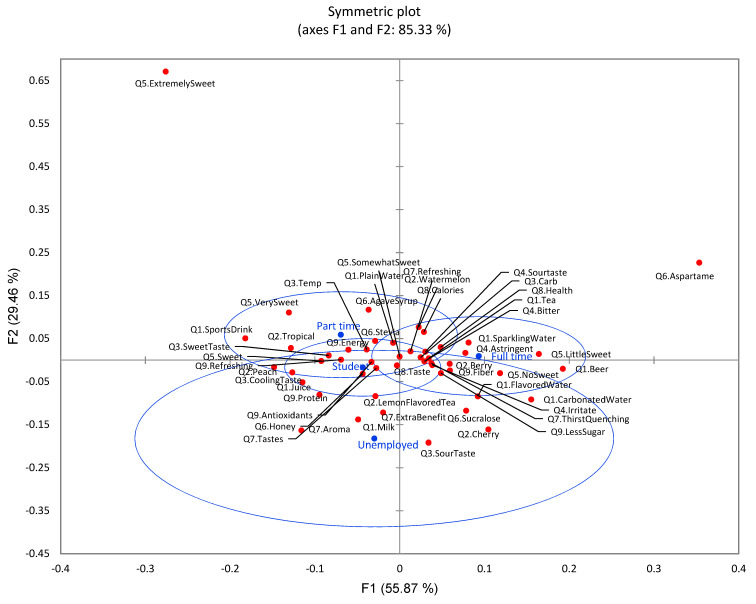
The first two dimensions of correspondence analysis (CA) symmetric plot using employment status (college student, full time, part time, and unemployed) as Rows and all nine survey questions as Columns (*n* = 901) (4 rows × 54 columns). Confidence ellipses on the plot were automatically created by XLSTAT software based on CA with observed *χ*^2^ = 163.41 and *p* = 0.389.

CA did not display a significant difference (*p* = 0.389) between four groups of employment status toward nine survey questions overall, although certain separation of groups was still identified (Figure 14). The full-time group preferred flavored water, teas, beer, carbonated water, and sparkling water with thirst-quenching functions, fiber, and less sugar. Part-time and student groups preferred sports drinks and juice with higher expectation of taste and functions of refreshing, energy, protein, and antioxidants. Considering flavored water sensory quality, the part-time and student group displayed higher preference on temperature (low temperature), cooling, and sweet taste, while the full-time employment group had more concerns with sour taste, bitter, astringent, and irritate mouthfeel. This was further confirmed that part-time and college students preferred sweet and very sweet taste, while the full-time group preferred little sweet and no sweet. Additionally, the full-time group had more concerns about health issues related to sugar, and preferred artificial sweeteners such as aspartame. The part-time and student groups were concerned about the taste of flavored water and preferred honey as a sweetener.

### 3.7. Consumer Segments—Pattern with Multiple Demographic Variables

Agglomerative hierarchical clustering (AHC) was conducted to group people based on the similarities in the patterns of their responses, assuming everyone in the same produced segment has similar preferences. The AHC analysis resulted in two consumer preference segments/clusters (dendrogram not shown). Cluster 1 had 410 participants (45.5% of the total), and cluster 2 had 491 participants (54.5%).

**Figure 15:**
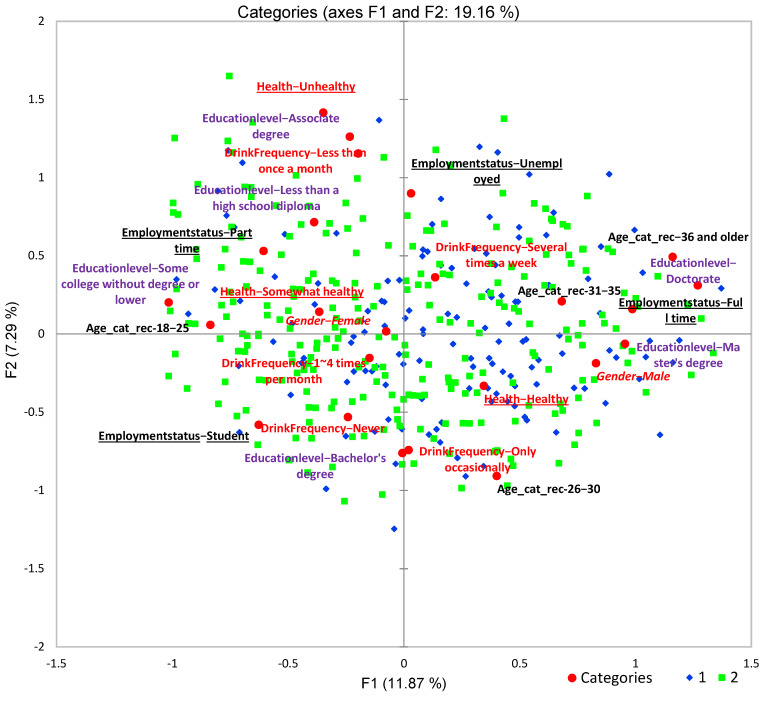
Multiple correspondence analysis (MCA) of two clusters of consumers (blue diamonds represent cluster 1 with *n* = 410 and green squares represent cluster 2 with *n* = 491) characterized by six demographics (gender, age, health condition, drink frequency, education level, and employment status) in different colors and fonts.

Demographic information for the overall panel and compared proportion of response frequencies between the two consumer segments/clusters is shown in Table 2. The two consumer segments had minimal differences for gender and consumption frequency, but more differences within groups of age, health, education level, and employment conditions were identified (chi-square analysis, *p* ≤ 0.05). To visualize the characteristics of demographic information for both consumer segments, MCA was conducted and shown in Figure 15. The population in cluster 1 contained older participants, especially for the age group of 36 and older. More participants in this segment rated themselves as healthy. They had a higher education level (master and doctoral degrees) and a full-time job, drinking flavored water less frequently (never and occasionally). In contrast, cluster 2 included more young participants (18–25 years old), and they rated themselves less healthy compared to the population in cluster 1. This segment contained populations who had lower education (mainly college students) and held part-time jobs or were unemployed.

**Figure 16:**
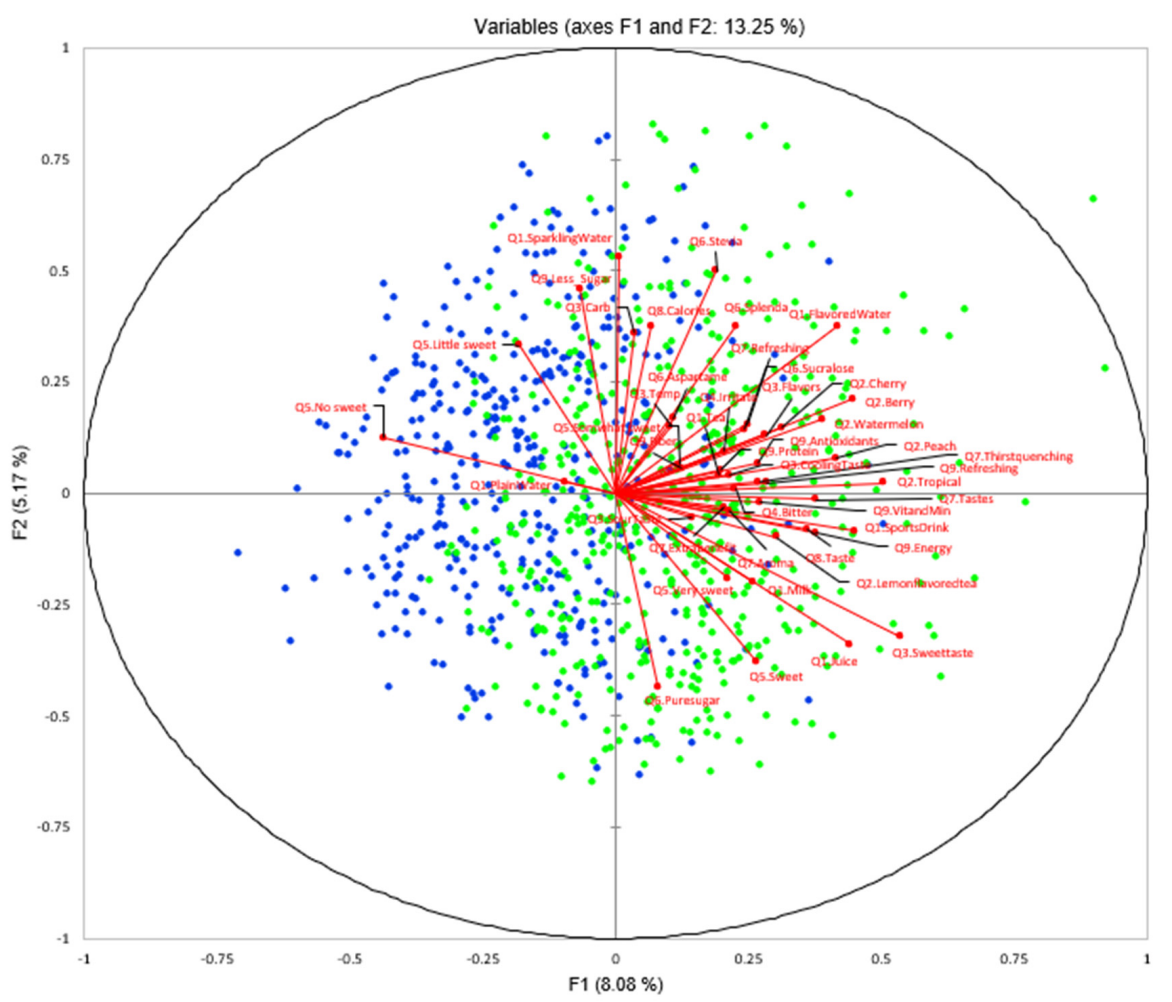
Internal preference mapping (IPM) of two clusters of consumers (blue diamonds represent cluster 1 with *n* = 410 and green squares represent cluster 2 with *n* = 491) characterized by nine survey questions (Q1–Q9, 41 selection total), which were selected by significance of chi-square analysis between two clusters.

Furthermore, the two consumer segments showed significant differences within each survey question (chi-square analysis, *p* ≤ 0.05, Table 1). A visualized biplot using IPM is shown in Figure 16. Respondents in segment 1 (cluster 1) exhibited higher preference to plain water, while the cluster 2 population preferred flavored water, sports drinks, and juice. Cluster 2 consumers had higher expectation of the flavored water functions such as thirst-quenching, refreshing, taste, energy, vitamin and mineral, and antioxidants, compared to respondents in cluster 1. Specifically on sensory quality of flavored water, respondents in cluster 2 had higher demand for flavored water temperature, sweet taste, and flavors, specifically for tropical, berry, watermelon, and peach flavors. In addition, respondents in cluster 2 had more concerns about off-notes such as bitterness taste and irritate mouthfeel of flavored water. For the attitude toward sugar, more respondents in cluster 2 preferred sweet and somewhat sweet taste of flavored water, while the respondents in cluster 1 preferred little sweet or no sweet. Consumer segment 1 was concerned about the taste of sugar-reduced flavored water; although they were also concerned about caloric content, preferring artificial sweeteners such as stevia, Splenda, and sucralose.

## 4. Discussion

Plain water was ranked in the top of drinks in this study, which is consistent with the literature; the transition is caused by consumer interest in healthier and more functional products [4]. Flavored water was ranked in the top four most popular drinks, which indicated that flavored water was well accepted and consumed on a regular basis. Flavored water, categorized as any water infused with flavors, is meeting this image. In general, consumers want flavored water to hydrate and offer an added functional benefit. Functional benefits are associated with health and well-being, offering added value and consumer appeal [1]. There is an increasing interest for the food industry to develop products that are perceived as being refreshing and high in nutritive value such as healthy plant waters for sport drinks.

In this study, the refreshing effect, thirst-quenching effect, and taste quality of flavored water were the three most important considerations regarding the satisfaction of consumer flavored water expectations. Refreshing has been defined as “serving to restore strength and animation, to revive, to arouse, to stimulate, to run water over or restore water to, with thirst-quenching properties” [17]. Refreshing perception is related to psychological and physiological enhancement such as thirst-quenching, rehydration, energizing, and mental energy enhancement [17]. Specific sensory properties of foods and beverages favoring refreshing perception have been identified. The simulation of refreshing perception can come from three different sensory dimensions including trigeminal, taste, and flavor. According to the definition of refreshing perception, thirst-quenching is intimately related to it. The results of consumer’s definition for refreshing perception in this study were consistent with the literature in that the primary function of flavored water is hydration or thirst-quenching [4], and the second most expected attribute for flavored water is thirst-quenching. Additionally, this study ranked taste as the third most expected flavored water attribute, demonstrating that flavor (aroma and taste) is always an important factor determining food consumption and repurchase.

In this study, the results of expected extra benefits to be added for flavored water further showed the importance of refreshing and was highly expected to be added. The result that participants expected vitamins, minerals, and antioxidants the most for extra benefits was coincident with recent beverage trends, indicating that consumers are looking for functional beverages capable of enhancing brain performance and health, beauty, gut health, and functional waters with added vitamin, mineral, herbs, and fruit [18]. Health effects in both ingredients and final products are essential to satisfy consumer preferences [18].

Sensory quality is key for flavored water consumption and this study indicated the importance of temperature and flavor. Cooling has been identified as a key sensory driver of refreshment [19]. Water at 5 °C is perceived as more thirst-quenching than warmer water at 22 °C [20]. In another study, 78% of participants listed cold to describe the sensory characteristics of refreshing foods and beverages [21]. Sensory characteristics related to cold temperature were consistently shown to have a positive influence on refreshing [19,22]. These literature results indicated an endogenous relationship between temperature, flavor, and refreshing perception.

Flavor and sweet taste, the second and third most highly ranked sensory qualities (Figure 4), were essential consumption determining factors for flavored water. This result was consistent with a survey from the literature, which shows flavor is the number one driver of flavored water purchases, and beverages possess the biggest flavoring application market share [18]. Human preference for sweet taste is universal, although hedonic responses toward sweet taste change over a lifetime [23]. In this study, the consumer preference toward sweet taste is in line with the current beverage industry trend in sugar reduction with compensation of intensive sweeteners. This trend arose initially because excess sugar consumption is linked to multiple adverse health conditions, and soft drinks are one of the largest dietary sources of added sugar [24].

In addition to sweet, the results of the popularity of lemon flavor in this study were consistent with the literature in that lemon is the most dominant flavor in the market, followed by orange [7]. Diverse flavors are available for flavored water in the market, but flavor diversification will continue alongside market share growth [6]. The results of disliking bitterness and other off-notes indicated the importance of sugar (sweetness) to beverages. Sugar not only provides a sweet taste, but also masks or reduces the perception of bitterness, astringency, and sourness [11]. It is a consensus that sweetness is innately sought after as an energy source for survival, while bitterness is associated with an aversion of alkaloids and other potentially deadly toxins found in nature [25]. Therefore, the findings of disliking bitterness, astringency, irritation, and sourness from this study indicate human nature.

Regarding consideration on choosing sugar-reduced flavored water, this study suggested that consumers preferred less sugar. This phenomenon might be associated with consumer awareness of the negative health effects caused by sugar over-consumption. Human preference for sweetness is natural, although the determinants of a person’s preference for sweetness are largely unknown [23].

The results for the specific factors that consumers consider for the reduced-sugar flavored water further confirmed the importance of taste or flavor. Even though consumers knew the issues of calories and negative health effects caused by sugar consumption, the taste/flavor could not be compromised. This indicated that an appealing sensory profile is demanded by consumers, despite the push for sugar reduction. Meanwhile, a portion of consumers would like to choose sugar-reduced flavored water/beverages because of the concerns of calories and health effects imparted by sugar consumption. These results showed consumers had a complex decision-making process influenced not only by rationale but also by habit without a conscious effort.

The findings of preference for natural sweeteners in this study were consistent with the current trend that consumers highly demand natural, healthy sweeteners [26], which, in turn, will shape the beverage industry to ensure that consumer demands for naturally sweetened products are met. Artificial sweeteners not only have many limitations in terms of sensory profile and stability in final products [26], but they are also associated with negative health effects such as toxicity and cancer [27].

For the flavored water expectation, flavor preference, and sugar reduction context investigated in this study, all six demographic variables had the potential to affect. Gender, age, and health condition were biological variables; flavored water drinking frequency belongs to psychological variables (behavioral variables), while education levels and employment status belong to the social aspects of demographic information.

The gender difference toward flavored water health benefits in this study was coincident with the literature that women generally show a healthier pattern of food choice [28]. Gender differences toward mineral water are observed by consumers in Brazil; one specific example would be the female consumer’s consumption of mineral water for hydration and well-being [14]. Regarding flavor, males more preferred lime flavor and had a higher tolerance toward bitterness and astringency in flavored water than females. This phenomenon may be tied to gender differences in bitter perception, with males being less likely to sense and accept bitterness [29,30]. Additionally, no significant difference in preference toward sugar-reduced flavored water was identified between genders in the current study, although it has been reported that males were more likely to like options higher in sweetness [31].

The finding that different drink preference between ages in this study was slightly different from the literature, which has reported that younger participants had a higher consumption share of water, milk, beer, and carbonated beverages [32]; however, the results might depend on how the age groups are defined. More population within younger generations expected extra benefits and energy functions of flavored water, which is coincident with the fact that energy drinks are typically marketed to and consumed by adolescents and young adults [33]. In addition, age has been shown to be a significant variable in food choice decisions, with acuity change cited as one of the key reasons [34]. The decreased sensory perception and appreciation of food and drink in the elderly may influence the flavor perception and the intake of food and beverages [13].

Regarding health condition, this study showed the healthy group preferred healthier beverages. Few studies have reported this phenomenon, and the relationship between them might be mutual cause and effect. The relationship between sweet taste and health has been of interest to researchers for many years; however, there are no clear conclusions. Obese people might have a lower taste sensitivity, and, consequently, a higher propensity for sweet foods [35]. Controversial results are reported as well [36]. Nevertheless, there is no clear relationship between sensitivity of sweet, salty, sour, or bitter tastes and weight status [37].

The flavored water drinking frequency demographic variable is a key factor influencing the expectation of flavored water and its associated sensory quality and sugar reduction, since frequent consumers have more knowledge and experience related to flavored water/beverages. This study indicated that habitual consumption of a food increases its acceptability, and is consistent with the literature [38]. In addition, higher consumption frequency might decrease taste sensitivity [30], and consequently preferring more flavor (aroma and taste) and less concern regarding off-tastes such as bitterness and irritation, as shown in this study.

For the education group, the more prevalent choice of beer in the higher education groups is controversial to the literature in that beer drinkers have a lower education level than non-beer drinkers [13]. The impact of participant education levels on food choice is hardly found in the literature. Education might influence people’s knowledge, consequently affecting attitude and behavior [39].

Employment might influence income and economic status, which determine purchase frequency and the type of beverages available to choose [40]. However, none of the literature has a sufficient explanation of the relationship between employment status and choice of beverages. More research on this topic is needed.

The above consumer demographic analysis was based on a single variable. It would be interesting to identify preference determined by multiple variances such as the interaction of these six demographic variables. In this study, according to consumer segment characteristics and their attitude and expectation toward flavored water, flavored water was mainly consumed by younger generations, especially college students. They had various expectations not only of flavored water functions but also flavors. They favored low temperature, cooling and sweet tastes, and diverse flavors. They also expressed sensitivity to bitterness taste and irritate sensation, which was seen alongside their concern with the taste of sugar-reduced flavored water. The finding of the consumer segment for flavored water can be used as basic data to establish marketing and sales strategies for promoting flavored water [10]. It should be pointed out that consumer segments in this study were solely based on demographic information. Other psychographic variables such as personality, values and beliefs, and lifestyle might also impact consumer expectation and attitude toward beverages [41]. In addition, although sample size was large for this study, we did not check how many participants were non-TWU individuals. The results might have bias for generalization to all consumers in the United States.

## 5. Conclusions

This study indicates that flavored water was ranked the fourth most popular drink. Refreshing functions, flavor, and sweet taste were the top three factors that consumers considered when they chose flavored water. Regarding flavor, lemon was the most popular, and consumers preferred sweetness (sugar), while bitterness was least preferred. Sugar reduction is a hot research topic further confirmed by participants’ caloric content concern. However, consumers concerned with taste for the sugar-reduced flavored water, with pure sugar and honey preferred as sweeteners. Reliance on pure sugar and honey in a sugar-reduced flavored water would be hard to accomplish in the food industry, since the common approach is to use sweetness substitutes such as high-potency sweeteners. Additionally, consumer segments, either by single-variable or multiple-variable patterns, were observed for the expectation of flavored water, specific sensory quality, and choice of sugar-reduced flavored water. The younger, low-education, less healthy participants expected various functions and flavors, especially preferring sweet taste; the older, educated, employed, healthy participants had the opposite expectation of flavored water.

The findings from this study provide consumer insight of flavored water, and the gained knowledge could be used in new flavored-water product design, especially for sugar-reduced drinks. The findings of flavored water consumer segments provide potentially new marketing strategies for different consumer segments. Although our original study was designed to focus on the female consumer, consumer segmentation by variables other than gender has potential limits of gender bias.

## Figures and Tables

**Table 1:** Survey questions, responses, and percentages for survey questions distributed between two clusters.

Expectation	Question	Response	Cluster 1 (%)	Cluster 2 (%)	Significance
Expectation on flavored water functions	Q1. What type of drinks/beverages do you usually drink? CATA	Plain water	94.4	88.8	*
		Flavored water	36.6	45.0	*
		Sparking water	40.5	28.1	*
		Tea	59.5	66.2	*
		Coffee	63.2	57.8	
		Beer	22.9	18.1	
		Carbonated water	25.1	21.4	
		Sports drink	11.5	33.0	*
		Juice	23.7	48.5	*
		Milk	18.8	38.1	*
		Other	15.9	15.1	
	Q7. Which effect would you expect the most from the flavored water/beverage? CATA	Thirst quenching	64.6	81.1	*
		Refreshing	80.7	93.1	*
		Extra benefit	13.9	20.0	*
		Tastes	58.0	71.7	*
		Aroma	8.8	13.0	*
		Other	2.2	0.0	*
	Q9. If you can buy the beverages with additional benefits, what benefits would you like it be added? CATA	Energy	37.3	66.8	*
		Vitamins, minerals	65.4	80.0	*
		Protein	23.4	36.0	*
		Fiber	16.1	24.6	*
		Refreshing	42.7	51.3	*
		Antioxidant	55.4	65.4	*
		Less sugar	49.5	39.3	*
		Other	2.4	0.0	*
Expectation on flavored water sensory quality	Q3. Which of the following factors do you consider the most when deciding flavored water/beverage to drink? CATA	Temperature	58.8	65.2	*
		Carbonation	42.4	33.2	*
		Cooling taste	30.5	39.1	*
		Sweet taste	27.1	64.4	*
		Sour taste	6.3	10.2	*
		Flavors	44.1	59.1	*
		Others	9.5	0.2	*
	Q2. Which types of flavored water/beverages do you consume the most? CATA	Lemon	50.0	53.4	
		Lime	33.4	29.1	
		Lemon flavored tea	13.9	26.7	*
		Tropical	15.1	39.5	*
		Berry	25.9	45.0	*
		Watermelon	8.3	22.2	*
		Peach	13.7	34.4	*
		Cherry	11.7	23.0	*
		Plain	44.9	41.1	
		Other	25.1	7.5	*
	Q4. Which of the following attributes that you do not expect of the flavored water/beverage? CATA	Sour taste	54.9	58.7	
		Bitter	73.7	85.7	*
		Astringent	64.9	70.9	
		Irritate	71.2	79.2	*
		Other	5.6	0.0	*
Expectation on flavored water sugar reduction	Q5. How much of sweetness do you like the most for your regular beverage consumption? SC	None sweet	24.4	5.1	*
		Little sweet	36.3	16.1	*
		Somewhat sweet	22.4	30.8	*
		Sweet	13.2	33.4	*
		Very sweet	3.2	13.6	*
		Extremely sweet	0.5	1.0	
	Q8. When you choosing sugar-reduced products, what factors do you consider the most important? CATA	Calories	53.2	60.7	*
		Cavities	17.1	21.2	
		Health	39.3	41.5	
		Taste	51.0	66.0	*
		Other	9.8	0.6	*
	Q6. Which sweeteners do you prefer to eat or drink with? CATA	Pure sugar	50.7	57.4	*
		Stevia	25.6	31.6	*
		Splenda	14.9	28.5	*
		Honey	52.2	56.2	
		Aspartame	2.2	10.0	*
		Sucralose	0.7	7.3	*
		Agave syrup	21.7	20.8	
		Other	6.8	0.6	*

CATA = check all that apply; SC = single choice. * indicates a significant difference in the percentages between the two clusters in each row category with chi-square analysis (*p* ≤ 0.05). Cluster 1: *n* = 410; Cluster 2: *n* = 491.

**Table 2:** Number and percentages on overall sample and by clusters for demographic variables of the survey.

Question	Response	Overall		Cluster 1		Cluster 2		Treatment for Statistical Analysis
		*n*	%	*n*	%	*n*	%	
Gender	Female	823	91.3	375	91.5	448	91.2	“Others” were not included in analysis
	Male	76	8.4	34	8.3	42	8.6	
	Others	2	0.2	1	0.2	1	0.2	
Age	18–25	440	48.8	159	38.8	281	57.2	“36–40”, “41–45”, “46–50”, and “51 and older” were combined to “36 and older”
	26–30	161	17.9	79	19.3	82	16.7	
	31–35	97	10.8	47	12.0	48	9.8	
	36–40	48	5.3	33	8.0	15	3.1	
	41–45	43	4.8	23	5.6	20	4.1	
	46–50	43	4.8	22	5.4	21	4.3	
	51 and older	69	7.7	45	11.0	24	4.9	
Reported health condition	Very unhealthy	10	1.1	5	1.2	5	1.0	“Very unhealthy” and “unhealthy” were combined to “unhealthy”; “healthy” and “very healthy” were combined to “healthy”
	Unhealthy	61	6.8	20	4.9	41	8.4	
	Somewhat healthy	369	41.0	152	37.1	217	44.2	
	Healthy	392	43.5	197	48.0	195	39.7	
	Very healthy	69	7.7	36	8.8	33	6.7	
Flavored Water drink frequency	Never	38	4.2	39	9.5	15	3.1	
	Only occasionally	176	19.5	90	22.0	95	19.3	
	Less than once a month	44	4.9	26	6.3	29	5.9	
	1~4 times per month	255	28.3	161	39.3	221	45.0	
	Several times a week	388	43.1	94	22.9	131	26.7	
Education level	High school or lower	35	3.9	11	2.7	24	4.9	“High school or lower” and “some college, no degree” were combined to “some college without degree”
	Some college, no degree	215	23.9	69	16.8	146	29.7	
	Associate degree	113	12.5	43	10.5	70	14.3	
	Bachelor’s degree	272	30.2	129	31.5	143	29.1	
	Master’s degree	182	20.2	105	25.6	77	15.7	
	Doctorate	84	9.3	53	12.9	31	6.3	
Employment status	College student	335	37.2	133	32.4	202	41.1	“Unemployed”, “self-employed”, “homemaker”, and “retired” were combined to “unemployment”
	Full time	329	36.5	172	42.0	157	32.0	
	Part time	193	21.4	83	20.2	110	22.4	
	Unemployed	19	2.1	10	2.4	9	1.8	
	Self employed	10	1.1	3	0.7	7	1.4	
	Homemaker	11	1.2	7	1.7	4	0.8	
	Retired	4	0.4	2	0.5	2	0.4	

*n* = number of participants; % = percent frequency. Overall sample: *n* = 901; Cluster 1: *n* = 410; Cluster 2: *n* = 491.

## Data Availability

Our data are available for review by request.
